# Surgeons and Surgery in Victorian Yeovil

**Published:** 1984-04

**Authors:** John R. Guy

**Affiliations:** Archivist at the Marsh-Jackson Postgraduate Medical Centre, Yeovil District Hospital


### Surgeons and Surgery in Victorian Yeovil

**John R. Guy, B.A., Ph.D., A.R.Hist.S.**
Archivist at the Marsh-Jackson Postgraduate Medical Centre, Yeovil District Hospital

### SURGEONS AND SURGERY IN VICTORIAN YEOVIL

The Yeovil General Dispensary, forerunner of the present District Hospital, first opened its doors in March, 1858. It was founded on the initiative of three medical practitioners in the area, ELIAS TAYLOR WARRY, RUSSELL ALDRIDGE and WILLIAM FORD BENNET, of whom two, Warry and Aldridge, were themselves native Yeovilians.

Warry had been a House Surgeon at St Bartholomew's Hospital, where he had trained towards the end of the long career of John Abernethy, who attracted large numbers of students to his lectures. He had begun his medical career in the 1820s (L.S.A. 1824, M.R.C.S. 1825) and subsequently gained an F.R.C.S. by examination in 1847, and an M.D. from the University of St Andrews in 1856, during the course of his medical practice in Yeovil.

Aldridge graduated M.D. from Edinburgh in 1854, submitting a thesis *On Tetanus*, a copy of which is now held in the Yeovil Hospital archives. After a spell as Senior Surgeon at the Birmingham and Midland Counties Lying-In Hospital, he returned to his native town, and practised in Yeovil until his death in 1895. He published a very short communication in the *B.M.J.* on 'The Use of Iron in Scarlatina' (Aldridge, 1871), in which he advocated 'the liquor of pernitrate of iron, in syrup or glycerine, in doses of ten minims every three hours for children of from one to six years, increasing, according to age, to fifteen, twenty-five or thirty minims'. He also recommended the use of 'warm fomentations to the neck' in cases of scarlatina anginosa.

Ford Bennet, the third of the Dispensary doctors, practised in Ilchester, and had had a varied career. He had been a surgeon in the Merchant Marine, Surgeon-Superintendent of the Indian Emigration Service, and had also worked as an army surgeon at the famous—or notorious—Scutari hospital with Florence Nightingale during the Crimean War.

Another retired military surgeon in Yeovil at this time was CHARLES DEANE STEELE, F.R.C.S. In 1854—the year in which Aldridge had submitted his thesis—Steele published an account of 'Two Cases of Extraction of Loose Cartilages from the Elbow Joint' (Steele, 1854). The reference was to injuries that he had treated in 1852 and 1853 as surgeon to H.M.S. Arethusa. Both cases are of interest, but for reasons of space, only one is summarised here.

> E.D., aged 21, ordinary seaman. At the radial side of ulna, just between the outer condyle of humerus and head of radius, where there ought to be a depression, a swelling was detected, and a hard substance could be felt. It was tender here on pressure, and pain, which was caused on extending the arm, radiated from this. The hard substance was very slightly moveable longitudinally, but not laterally; on flexing the arm it sank in so as barely to be felt, and on extending it again it protruded; and if this were done suddenly, there would be a loud snap on the joint, with considerable pain . . .
>
> He was put under the influence of chloroform, and a longitudinal incision was made down on the foreign body. On exposing it, and lifting it through the opening in the synovial membrane, it was found to be adherent by one side, which had to be divided ere it could be removed. It was white and glistening, of the size of a small hazel-nut; there was a rough spot on one side, as if it had recently been broken off from an adhesion, and on this side there was a sort of pellicle, where it adhered before it could be separated from the joint. The wound was closed accurately by three sutures, and isinglass-plaster intervening between the stitches; over all a large piece of gold-beaters' skin; then two pads of lint, so as to press the sides of the wound together; over all a bandage. It was then fastened to a well-padded angular splint.

Not the least interesting features of Steele's cases, which are carefully described, are the use of chloroform as anaesthetic aboard one of H.M.'s ships in 1852, only about six years after its first use in English surgery, and the account of his practice in wound-closure, with the use of gold-beaters' skin as an inert layer between the stitches and the lint, preventing adhesion of the one to the other.

Orthopaedics was also an interest of GEORGE FLOWER, M.R.C.S., appointed Consulting Surgeon to Yeovil District Hospital in 1880. In November 1895 Flower published an account of 'A Case of Dislocation of the Femur on to the Pubes, Fracture of the Neck, and Removal of the Head of the Bone'. (Flower, G. J. W., 1895) It is a good illustration of the kind of heroic surgery that men like Flower were prepared to undertake, on this occasion in the patient's home and not in the hospital. The following is an extract from his paper.

A. H., aged 48, who had been under my care for eight or nine years, suffering from locomotor ataxy... In stepping off the pavement had fallen and dislocated his hip, the head of the femur being forced onto the pubes. All immediate attempts at manipulating the bone into place having failed... [ether] was given [and] manipulation recommenced... Suddenly, as the limb was being rotated, the head of the bone separated with a snap from the shaft, which then assumed its proper axis, leaving the head of the bone under the femoral vessels, so that the femoral artery could be felt pulsating over it. The limb immediately presented a dusky—I might almost say purple—hue, from venous congestion, and became cold. It was obvious that the only possible course was to cut down upon and remove the head of the bone; and this was accordingly done by means of an incision of about five inches in length, and just to the outer side of the vessels. The anterior crural nerve and the femoral vessels were turned aside, and the head of the bone was removed with the lion forceps. The incision was closed with chromic gut sutures, dressed with iodoform and double cyanide gauze, and a Desault's splint applied.

Subsequently, said Flower, the patient walked 'as well, if not better, than he did before it happened'. George Flower was succeeded as a surgeon to Yeovil District Hospital by his son Norman, who was himself to publish a couple of interesting papers (Flower, N., 1911: 1913) one on a case of Apyrexial Typhoid in Yeovil in 1913.

One of the earliest surgeons on the staff of the first Yeovil District Hospital when it opened in 1872 was EDWARD CHARLES GARLAND, M.R.C.S., who had succeeded Warry as surgeon to the Dispensary when he retired in 1864. Garland was M.O.H. for Yeovil, and associated with its hospital as Surgeon, Consulting Surgeon and as Vice-President until his death in 1903. He had served on the staff of the Royal Sea-Bathing Infirmary at Margate and at the Southern Hospital in Liverpool, after taking his M.R.C.S., L.M. & L.S.A. in 1855.

Garland's particular interest was in obstetrics, and whilst at Liverpool he had published a report of a case of placenta previa. (Garland, 1858). From the period of his Yeovil practice dates an account of 'A Case of Dermoid Ovarian Tumour, Escaping per Rectum'. (Garland, 1866).

Mrs A. B., aged 25, supposedly 5 months pregnant. She was suffering from great debility and experienced uneasiness hardly amounting to pain in the iliac region, where there was an amount of fullness on the left side. For some time she had suffered from diarrhoea.

Upon examination of the motions very little faeces appeared, but there was a considerable quantity of very offensive purulent matter. This discharge of purulent matter, occasionally amounting to even pints at a time, continued until October, when a large tuft of hair was found protruding from the anus. Slight traction was made but as it gave pain of course it was not continued; upon passing the finger nothing could be felt, but the hair was loosed at the side of the rectum some few inches up, a ligature was passed around and the external portion removed... Upon application of the speculum the hair could be seen protruding through an ulcerative opening of the rectum large enough to admit the finger, and it was decided to leave matters as they were. Some short time after a considerable homogeneous mass mixed with hair passed, the matter continuing to pass in larger quantities until June 1861, when hair again appeared, a few hairs at a time, and this continued occasionally until June, 1863...

In June 1863 Garland received this note from his patient 'I feel glad to be able to tell you that I think something has passed today which will throw light on all my illness. It seems as far as I can judge to be the not properly developed head of a child, hair is growing from it and there are two substances very like teeth attached to it. It gave me a little pain while passing...'

Garland, in association with Dr Tyler Smith, concluded that the tumour 'must have passed by suppuration slowly into the rectum. It was discharged partly piecemeal, and partly in the mass last alluded to.'

PTOLEMY AUGUSTUS COLMER, M.R.C.S. became Surgeon to Yeovil District Hospital in 1893. He was a member of a prominent local medical family. His father and namesake was a Yeovil doctor, who also served for a period as Mayor of the town. His brother, Robert Jacob, had a varied medical career, including a period as Medical Officer to the Ashanti Goldfield Corporation at Obuassi in West Africa, and as Surgeon on the 'Alpha', hospital ship of the Mission to Deep Sea Fishermen. He was on board the 'Alpha' during what became known as the 'Dogger Bank Outrage' when Russian warships fired on the fishing fleet, and he subsequently gave evidence at the International Enquiry into the incident in Paris.

In 1894 Ptolemy Colmer reported a case which had the distinction of being published in two medical journals. (Colmer, 1894; 1895).

F. T., a lad of $7\frac{1}{2}$. On September 30th appeared poorly, vomiting and complaining of pain in his abdomen. His mother put this down to a bilious attack, and administered castor oil, a dose repeated on the following day. On October 2nd, the boy being no better, she went to a chemist, and he prescribed a powder (calomel 1 gr., sugar 6 grs.) which was immediately vomited. This proving ineffectual, a second powder was given in the evening. On the following morning he appeared better, and took a cup of tea and milk about 7.30., immediately after which he wanted to have the use of his bowels, and was taken out of bed and placed on a chamber, when he passed a little fluid motion, and was placed back in bed. He died about four minutes later.

Colmer performed the post-mortem. He reported

'On opening the abdomen there was signs of recent general peritonitis which were especially marked around the caecum, the vermiform appendix was much thickened, and enlarged and presented a perforation through which was projecting a sharp point, and within which could be felt a hard body. On opening the appendix I found a body much resembling a date stone and apparently composed of hardened faecal matter from one end of which was protruding a sharp point, evidently that of a pin.'

Finally in this brief survey of surgeons and surgery in Victorian Yeovil, something must be said about arguably the most distinguished, WILLIAM ALFRED HUNT, M.R.C.S. Hunt was trained at King's College Hospital in London, where he was for a time dresser to Sir William Fergusson, the professor of surgery, who has been called the greatest practical surgeon of his day. Hunt who was to be a tireless publisher of papers throughout most of his career, produced his first as a student (Hunt, 1867) on 'Foetal Peritonitis in Utero'. But his passion was for oral surgery, and he became the first Surgeon-Dentist to Yeovil District Hospital. Although initially continuing in general practice, he eventually specialised almost exclusively in dental surgery. He was a man of inventive genius, and rose to become President of the British Dental Association. For one thing in particular he deserves recognition.

It was dentists who pioneered the use of anaesthetics. The American William Morton used ether at an operation at the end of 1846, and its first recorded use in England was by the London dentist James Robinson on 19th December 1846—two days before Liston's historic first surgical procedure with anaesthetic. Robinson was a close friend of Hunt's father, the Yeovil dentist William Hunt, who was dental officer to the original General Dispensary. Hunt senior is acknowledged as the first dentist outside of London to use a general anaesthetic for tooth extraction. Hunt junior was as much an experimenter and pioneer as his father. He published the first paper in this country on the extraction of teeth under local anaesthesia induced by the injection of cocaine (Hunt, 1886).

Karl Köller had demonstrated the use of cocaine as a local anaesthetic in ophthalmology in September, 1884. Hunt learned of this from his friend the great English ophthalmologist Edward Nettleship. During 1885 Hunt used cocaine administered by hypodermic injection to induce local anaesthesia prior to tooth extraction, and published his findings on 1st January 1886. There seems little doubt that the first use of general anaesthesia in dentistry outside of London and the first use of cocaine as a local anaesthetic for a similar purpose in England both took place in Yeovil by a father and son. Teaching hospitals are not the only centres of research and excellence!

### REFERENCES

ALDRIDGE, R. (1871) The Use of Iron in Scarlatina. *British Medical Journal,* 12 August 1871, 175.

COLMER, P. A. (1894:1895) An interesting case of Typhlitis and Peri-Typhlitis, with General Peritonitis ending in sudden death. *The Medical Press,* 7 October 1894, 395. Perforation of Appendix caeci by a pin; general Peritonitis, ending in sudden death. *The Lancet,* 23 March 1895, 745.

FLOWER, G. J. W. (1895) A case of dislocation of the femur on to the pubes, fracture of the neck, and removal of the head of the bone. *British Medical Journal,* 2 November 1895, 1101.

FLOWER, N. (1911:1913) Evidence of recent severe illness afforded by the nails. *British Medical Journal,* 15 April 1911, 869–870. Apyrexial Typhoid. *British Medical Journal,* 8 February 1913, 270.

GARLAND, E.C. (1858) Report of a case of Placenta Praevia. *The Lancet,* 11 September 1858, 280.

GARLAND, E. C. (1866) A case of Dermoid Ovarian Tumour, escaping per Rectum. *Medical Mirror,* March 1866, 143–145.

HUNT, W. A. (1867) Foetal Peritonitis in Utero. *Transactions of the Obstetrical Society of London.* 9, 15–16.

HUNT, W. A. (1886) Suggestions on the Use of Cocaine in Dental Surgery. *Journal of the British Dental Association,* 1 January 1886, 18–20.

STEELE, C. D. (1854) Two cases of extraction of loose cartilages from the elbow joint. *Medical Times Gazette,* 1854, 391.

This paper is based on that read at the Spring Meeting of the Surgical Club of South West England held at Yeovil District Hospital 29th and 30th April, 1983.

